# Prognostic Impact of Pretreatment Thrombocytosis in Epithelial Ovarian Cancer

**DOI:** 10.4103/njcp.njcp_134_19

**Published:** 2020-08

**Authors:** KS Okunade, O Dawodu, M Adenekan, CM Nwogu, O Awofeso, AO Ugwu, O Salako, S John-Olabode, OF Olowoselu, RI Anorlu

**Affiliations:** 1 Department of Obstetrics and Gynaecology, College of Medicine, University of Lagos, Lagos, Nigeria; 2 Department of Obstetrics and Gynaecology, Lagos University Teaching Hospital, Lagos, Nigeria; 3 Department of Anatomic and Molecular Pathology, College of Medicine, University of Lagos, Lagos, Nigeria; 4 Department of Radiotherapy and Radiation Oncology, Lagos University Teaching Hospital, Lagos, Nigeria; 5 Department of Haematology and Blood Transfusion, College of Medicine, University of Lagos, Lagos, Nigeria

**Keywords:** Nigeria, overall survival, platelets, progression-free survival, thrombocytosis

## Abstract

**Aims:**

This study was aimed at investigating the prognostic impact of pretreatment thrombocytosis in epithelial ovarian cancer (EOC) patients in Lagos, Nigeria.

**Methods:**

This was a retrospective cohort study involving the review of the clinical record of 72 patients with histologically confirmed EOC who were managed at the Lagos University Teaching Hospital, Lagos, Nigeria over a 7-year period from January 2010 to December 2016. Information on the sociodemographic data and platelet counts at diagnosis of EOC were retrieved from the patients’ medical records. Descriptive statistics were then computed for all baseline patients’ characteristics. Survival analyses were carried out using the Kaplan-Meier estimates. Multivariate analysis of these data was performed with the Cox proportional hazards model.

**Results:**

This study revealed that the prevalence of pretreatment thrombocytosis was 41.7% among the women with EOC. Fifty-three (73.6%) of the women had the advanced-stage disease (FIGO stage III-IV) while 52 (72.2%) had high-grade disease (II-III). The majority (66.7%) of the women had a serous histological type of EOC while 76.4% had documented recurrence. Pretreatment thrombocytosis was significantly associated with the women’s parity (*P* = 0.009), serum carbohydrate antigen 125 levels (*P* = 0.018), median progression-free survival (PFS) (*P* < 0.001), 3-year median overall survival (OS) (*P* < 0.001), type of primary treatment (*P* = 0.002), extent of cytoreduction (*P* < 0.001), presence of ascites (*P* = 0.002), International Federation of Gynecology and Obstetrics (FIGO) stage (*P* = 0.008), and histological type (*P* = 0.011). Pretreatment thrombocytosis was negatively associated with PFS (hazard ratio [HR] = 0.25; 95% CI 0.83, 0.75; *P* = 0.014) and 3-year OS (HR = 0.03; 95% CI 0.03, 0.27; *P* = 0.002).

**Conclusions:**

The study suggests that pretreatment thrombocytosis may be a useful predictor of survivals in EOC patients.

## Introduction

Ovarian cancer is the sixth most common cancer among women worldwide.^[1]^ There are almost 300,000 new cases of ovarian cancer diagnosed with approximately 180,000 deaths per year.^[1]^ Several studies from Africa and Nigeria have shown that ovarian cancer is the second most common gynecological cancer^[2–5]^ constituting about 7–8.2% of all gynecological malignancies^[6,7]^ and accounting for the second commonest cause of deaths among women admitted on the gynecological ward of a teaching hospital in Lagos.^[6]^ It is the leading cause of deaths in women with gynecological malignancies worldwide.^[8]^ In Nigeria, over 70% of ovarian cancer patients were diagnosed at an advanced stage.^[7,9,10]^

Lack of early predictive biomarkers is responsible for the high mortality rate. Until now, the most widely used biomarker for monitoring of ovarian cancer is carbohydrate antigen 125 (CA-125)^[11]^ with approximately 83% of the patients at advanced stage having CA-125 levels >35 U/mL.^[12]^ However, CA-125 is also elevated in a small proportion of people with endometriosis, pelvic inflammatory disease, pregnancy, hepatic cirrhosis, acute heart failure, tuberculosis, pancreatic cancer, lung cancer, liver cancer, and so on.^[13,14]^ Therefore, it has a relatively low positive predictive value and is usually not considered as an independent predictor.^[15]^ Thus, the effort to find other reliable and cheaper biomarkers that could be used alone (in resource-limited settings) or in combination with CA-125 for the effective evaluation and prognostic prediction of ovarian cancer is long-expected. The eminent role of platelet, a ubiquitously available parameter in regions with limited economic resources, has previously been discussed in the diagnosis of pelvic mass^[16]^ and, therefore, its impact on prognosis also needs to be examined.

Platelets are highly reactive cellular orchestrators of primary hemostasis, immunity, and inflammation^[17]^ and it is widely believed that platelets may play important roles in cancer growth and metastasis. The finding of thrombocytosis in patients with solid tumors was first made over a century ago.^[18]^ Incidentally, almost 40% of persons found to have thrombocytosis in the absence of iron deficiency and benign inflammatory conditions have occult malignant neoplasia including ovarian cancer.^[19]^ Experimental evidence had suggested that platelets actively promote cancer progression through different mechanisms and these may include shielding of cancer cells from immune attacks and stimulation of angiogenesis.^[20]^ A recent systematic review and meta-analysis conducted by Ye *et al.* reported that pretreatment thrombocytosis was a significant negative predictor of both survival in women with epithelial ovarian cancer (EOC).^[21]^ However, it is still not clearly understood whether prognosis in ovarian cancer patients is directly affected by the presence of a high level of platelets or if thrombocytosis is a surrogate for other prognostic indicators, such as advanced-stage disease, suboptimal cytoreduction, or even medical comorbidity. On this basis, the current study, therefore, investigated the impact of pretreatment thrombocytosis on progression-free survival (PFS) and overall survival (OS) in black Nigerian women with EOC.

## Subjects and Methods

### Study design and setting

This was a retrospective cohort study involving the review of case records of all histologically confirmed EOC patients managed at the Lagos University Teaching Hospital (LUTH), Lagos, Nigeria over a 7-year period from January 2010 to December 2016. LUTH is the largest tertiary institution that provides services to patients in Lagos and from the neighboring Southwestern states. The hospital is located in the central Lagos metropolis and offers mainly clinical services among which include gynecological oncology services.

### Eligibility criteria

Eligible participants were women with histologically confirmed EOC. Further eligibility criteria included those who commenced treatment within 6 weeks of diagnosis. Patients with non-EOC; those who failed to complete treatment or yet to complete the treatment at least in the last 3 years of the review period were excluded from the study.

### Data collection

The registration numbers of all women with EOC who were managed during the period under review were obtained from the gynecological oncology ward registers and the patients’ case notes were subsequently retrieved from the medical records department. Relevant information such as sociodemographic characteristics, menopausal status, body mass index (BMI), presence of major comorbidity (hypertension/diabetes mellitus/kidney disease), pretreatment platelets counts and serum CA-125 levels, type of primary treatment (primary debulking or neoadjuvant chemotherapy), extent of cytoreduction (optimal or suboptimal), presence of ascites (≥500 mL), FIGO stage of disease, histological subtype of tumor, and tumor grade were extracted using a standardized pro forma.

### Exposure and confirmatory outcome variables

Platelet counts at diagnosis of EOC were evaluated in all patients treated in the hospital and for whom there were complete follow-up data on PFS and 3-year OS. PFS was defined as those women without any clinical, biochemical, or radiological evidence of the disease within the first 36 months of completion of treatment. Evidence of the disease was defined clinically as presence of a pelvic tumor on bimanual pelvic examination; and/or biochemically as abnormal (>35 IU/mL) or increasing serum CA-125 level; and/or radiologically as the presence of a new pelvic tumor on abdominopelvic USS or CT scan. The 3-year OS was defined as those women who were still 36-month alive after completion of treatment. The endpoints for statistical analysis were 3-year OS and PFS while the exposure variable is elevated pretreatment platelet counts (thrombocytosis).

### Statistical analysis

All relevant data were analyzed using SPSS version 23.0 statistical package for Windows manufactured by IBM Corp., Armonk, NY, United States. Descriptive statistics were then computed for all baseline patients’ characteristics. Characteristics of patients were described by mean and SD (if normally distributed) or median and percentiles (if not-normally distributed) for continuous variables and by frequencies and percentages for categorical variables. PFS and OS of patients with normal platelet counts (150–450 × 10^9^/L) were compared with that of patients with thrombocytosis (>450 × 10^9^/L) using the Mann-Whitney U test. Survival analyses were carried out using the Kaplan-Meier estimates and statistical significance was determined by the log-rank test. Patients who were alive at last follow-up or those without recurrence were censored. Multivariate analysis of these data was performed with the Cox proportional hazards model. All testing was two-sided, and a *P* value of less than 0.05 was considered to indicate statistical significance.

### Ethical approval

Ethical approval for the study was obtained from the Health Research and Ethics committee of the LUTH (Approval number—ADM/DCST/HREC/1912) prior to the review of case records and data collection. Ethical principles according to the Helsinki declaration were considered during the course of the study.

## Results

On review of the case records of the 81 EOC cases managed in the hospital during the study period, only 72 cases had their complete clinical data available or eligible for final analyses. Excluded from the final analyses were two women who did not have documented pretreatment platelet counts, four women who failed to complete their primary treatment, and three women who were lost to follow-ups. The baseline characteristics of the study cohorts are shown in Table 1. The majority of the patients were postmenopausal (54.2%) and more than 50 years of age (66.7%).

The median (interquartile range [IQR]) platelet count was 428 (301–503) × 10^9^/L and 30 (41.7%) women had pretreatment thrombocytosis. Fifty-three (73.6%) women had the advanced-stage disease (FIGO stage III-IV) while 52 (72.2%) had high-grade disease (II-III). The majority of the cases of EOC were of serous histological type (66.7%). Fifty-five (76.4%) of the women had documented recurrence and 27 (37.5%) deaths were documented. The median PFS and 3-year OS were 15.0 (IQR: 8.0–29.5) and 36.0 (IQR: 23.0–36.0) months, respectively.

Table 2 shows comparison of the clinicopathological characteristics of patients with and without pretreatment thrombocytosis. Pretreatment thrombocytosis was significantly associated with the patients’ parity (*P* = 0.009), serum CA125 levels (*P* = 0.018), median PFS (*P* < 0.001), 3-year median OS (*P* < 0.001), type of primary treatment (*P* = 0.002), extent of cytoreduction (*P* < 0.001), presence of ascites (*P* = 0.002), FIGO stage (*P* = 0.008), and histological type (*P* = 0.011). There were no statistically significant association with age (*P* = 0.782), BMI (*P* = 0.052), menopausal status (*P* = 0.341), comorbidity (*P* = 0.963), and disease grade (*P* = 0.075).

**Figure 1:**
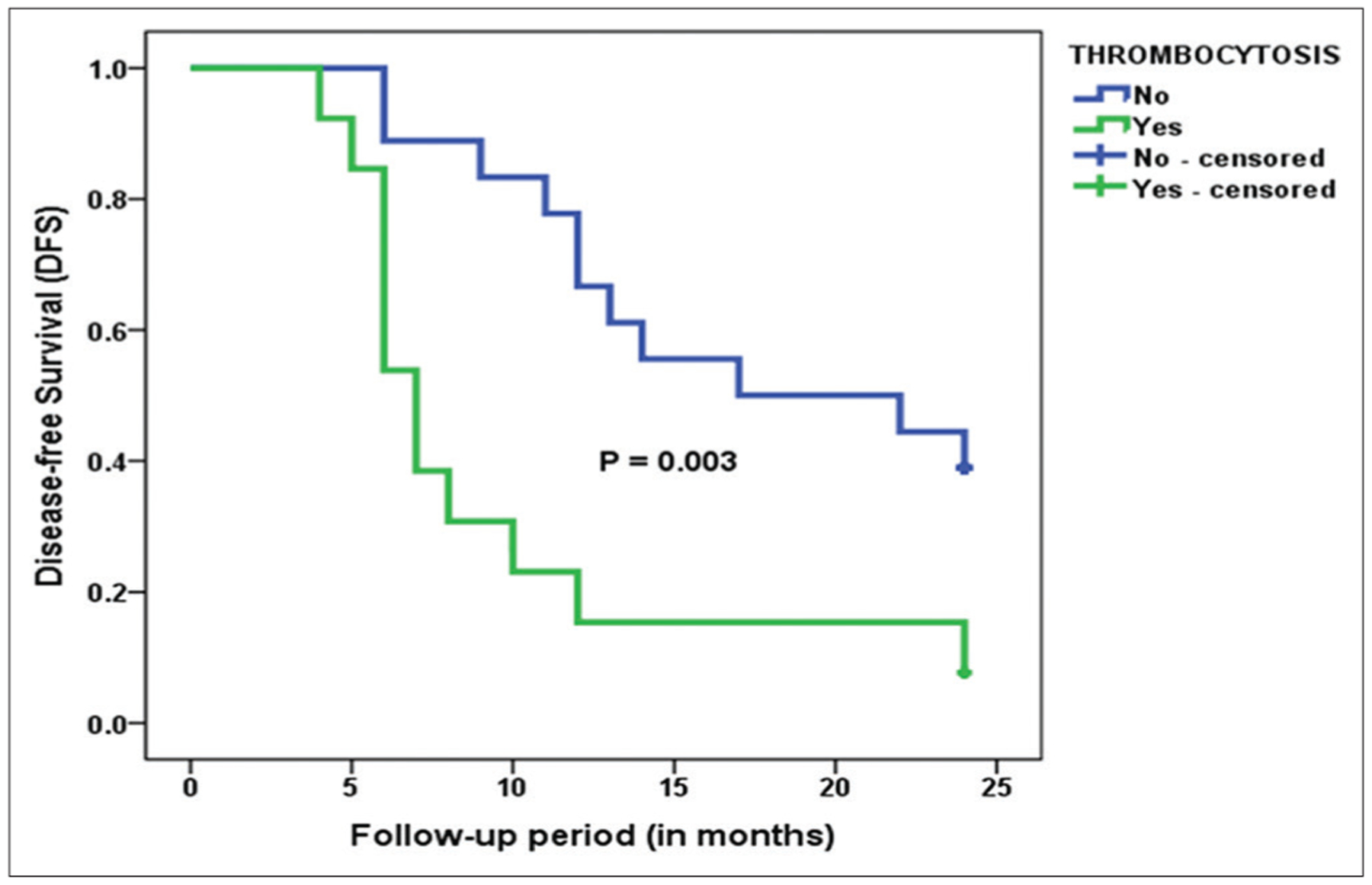
Kaplan-Meier curve of progression-free survival (PFS) stratified by thrombocytosis—Pretreatment thrombocytosis was associated with a shorter PFS (*P* = 0.003)

**Figure 2:**
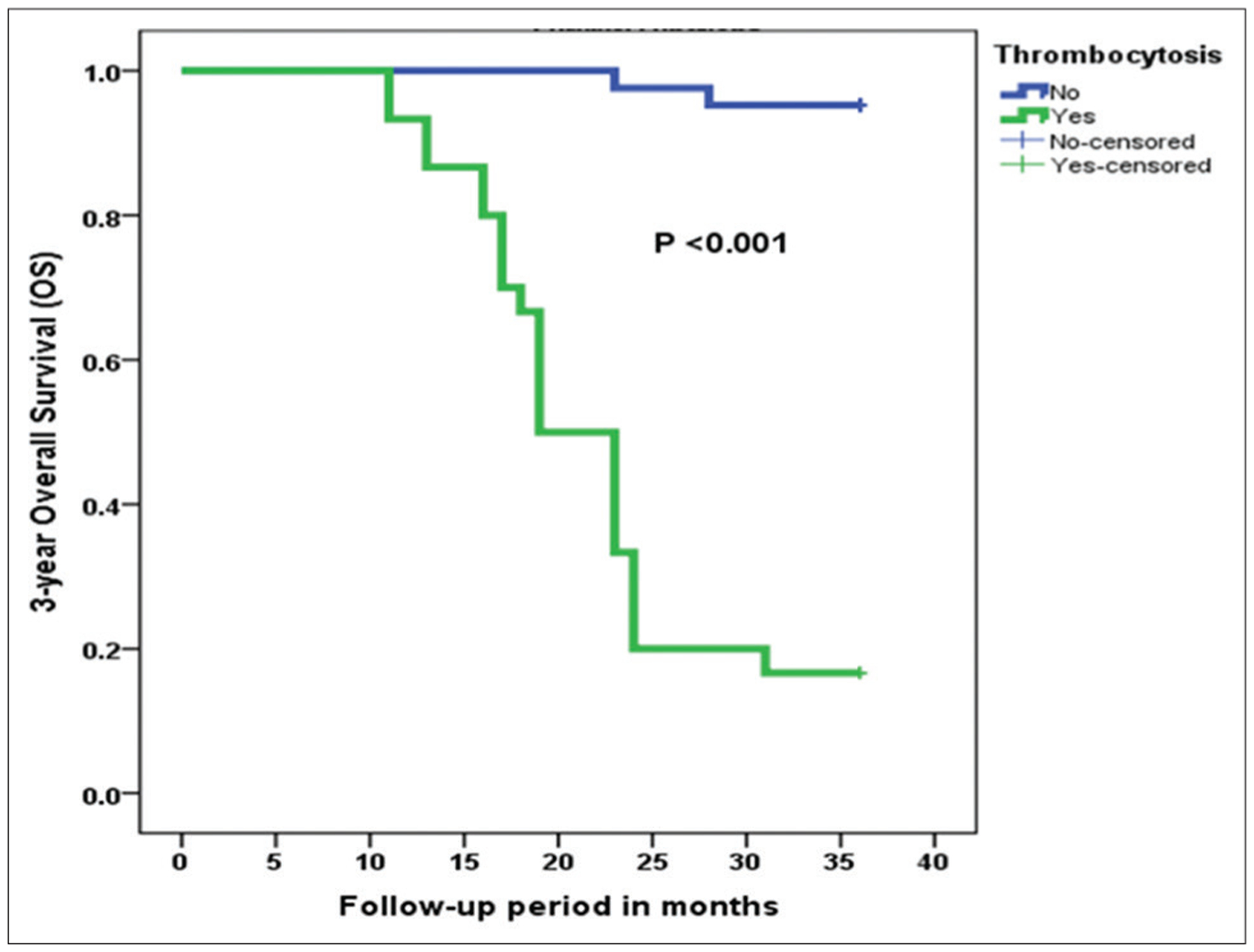
Kaplan-Meier curve of 3-year overall survival (OS) stratified by thrombocytosis—Pretreatment thrombocytosis was associated with a shorter OS (*P* < 0.001)

Figure 1 states the PFS while Figure 2 states the 3-year OS. Pretreatment thrombocytosis was associated with a shorter PFS (*P* < 0.001) and 3-year OS (*P* < 0.001). Multivariate analysis using Cox-regression statistics revealed that pretreatment thrombocytosis was an independent predictor of PFS (hazard ratio [HR] = 0.25; 95% CI 0.83, 0.75; *P* = 0.014) [Table 3] and 3-year OS (HR = 0.03; 95%CI 0.03, 0.27; *P* = 0.002) [Table 4].

## Discussion

This study is investigating the prognostic impact of pretreatment thrombocytosis in black Nigerian women with EOC. The majority of the patients were postmenopausal (54.2%) and this was higher than the proportion (40.0%) reported in a study conducted by Odukogbe *et al*. in Ibadan^[9]^ and our previous study conducted in the same setting in Lagos.^[7]^ This is, however, not unexpected as this current study only reported cases with histological diagnosis of EOC, which is commoner among older women, unlike our previous study that involved all cases of ovarian cancer in its final analysis.^[7]^

We reported that about 41.7% of our study cohorts in this study had pretreatment thrombocytosis and this is slightly higher than the 31.0% and 22.3% reported by Stone *et al*.^[22]^ and Allensworth *et al*.,^[23]^ respectively in the United States. This may be because of the racial differences in platelet counts as reported by Saxena *et al*.^[24]^ in their study of platelet counts in three racial groups where they indicated that black women had significantly higher platelet counts than their Caucasian counterparts. However, a much lower proportion of thrombocytosis (13.8%) was reported by Feng *et al*.^[25]^ in their study in China, and this apart from the reason adduced above may also be attributed to this study involving only women with high-grade serous ovarian cancer. The mechanisms of thrombocytosis in ovarian cancer and the role that this plays in abetting cancer growth are, however, still unclear.^[22]^

We demonstrated in this study that pretreatment thrombocytosis was associated with some clinicopathological characteristics and poorer survival in the Nigerian female population with EOC. We found that thrombocytosis was associated with the stage and histological type of the tumor, type of primary treatment, the extent of cytoreductive surgery and the presence of ascites at the surgery. These findings are consistent with several published studies involving patients with EOCs regardless of histological type^[22,23,26–28]^ and this indicates that platelet counts tend to increase concurrently with tumor progression and metastasis. Stone *et al*.^[22]^ reported that patients with thrombocytosis were significantly more likely to have advanced-stage disease and higher pretreatment levels of serum CA-125 than those with normal platelet counts. Ma *et al*.^[26]^ also found that EOC patients with thrombocytosis have a greater likelihood of having suboptimal cytoreduction.

Previous studies outside the African continent have suggested that platelet count and fibrinogen levels can serve as potential prognostic indicators in ovarian cancer patients.^[22,23,25–29]^ It was postulated that once the coagulation system is activated, it can directly or indirectly promote disease progression and metastasis with a resultant poorer prognostic outcome.^[30,31]^ Consequently, we were able to confirm in the current study that pretreatment thrombocytosis is an independent negative predictor of PFS and OS in Nigerian women with EOC. These findings were also confirmed in a recent systematic review and meta-analysis of 11 pooled studies conducted by Ye *et al*.^[21]^ reported that pretreatment thrombocytosis was a significant negative predictor of both PFS and OS in women with EOC for all stages and degrees of differentiation. This is at variance to the work of Allensworth *et al*.^[23]^ reported that thrombocytosis is not independently correlated with survival even after stratifying according to the FIGO stage. Such stratification was, however, not carried out in our current study.

The major limitation of this study was its retrospective design that depended on effective documentation of patients’ history with the potential for missed data. The poor record-keeping in our center also resulted in the unacceptably high number of EOC cases with incomplete clinical data which limited the follow-up period in the study to 3-years instead of the standard 5-years for assessment of survivors and subsequently the small sample size used. Additionally, our center is a foremost tertiary referral hospital in Nigeria and thus the majority of the patients seen with ovarian cancer have advanced disease and this may not be a fair representation of the general population. However, this study is a pilot research effort among women with ovarian cancer in Nigeria and therefore the preliminary data generated will form the basis for a future robust longitudinal study.

## Conclusions

This study revealed a high proportion of thrombocytosis among black Nigerian women with EOC. It further suggested that preoperative thrombocytosis reflects tumor burden and may also be a predictive determinant of survival in EOC patients. We, therefore, suggest that preoperative platelet levels may have value in counseling patients with ovarian cancer about their prognosis and the need for a more intense follow-up monitoring, prior to their primary treatment.

## Figures and Tables

**Table 1:** Baseline characteristics of study cohorts (*n*=72)

Variable	Frequency	Percentage
Age, in years		
<50	24	33.3
≥50	48	66.7
Mean age±SD=54.6±10.7 years		
Menopausal status		
Premenopausal	33	45.8
Postmenopausal	39	54.2
Parity		
Low parity (≤1)	23	31.9
High parity (≥2)	49	68.1
Median parity (interquartile range [IQR]) = 2.0 (1.0, 4.0)		
Body mass index (BMI), in kg/m^2^		
≤25.0	37	51.4
>25.0	35	48.6
Median BMI (IQR) = 24.7 (22.1, 28.9) kg/m^2^		
Co-morbidity		
No	55	76.4
Yes	17	23.6
Pretreatment CA-125 levels, in U/mL		
<250	25	34.7
≥250	47	65.3
Median CA125 (IQR) = 437.0 (144.0, 924.5) U/mL		
Type of cytoreductive surgery		
Optimal	37	51.4
Suboptimal	35	48.6
Presence of significant ascites		
No	37	51.4
Yes	35	48.6
FIGO stage		
Early (I-II)	19	26.4
Advanced (III-IV)	53	73.6
Tumour grade		
Low grade (grade I)	20	27.8
High grade (grade II-III)	52	72.2
Histological type		
Serous	48	66.7
Non-serous	24	33.3
Type of primary treatment		
Primary debulking	39	54.2
Neoadjuvant chemotherapy	33	45.8
Thrombocytosis		
No	42	58.3
Yes	30	41.7
Median platelet counts (IQR) = 428 (301, 503) × 10^9^/L		
Recurrence within 36 months		
Yes	55	76.4
No	17	23.6
Median PFS (IQR) = 15.0 (8.0, 29.5) months		
Vital status at 36-months		
Dead	27	37.5
Alive	45	62.5

Median OS (IQR) 36.0 (23.0, 36.0) months. SD: standard deviation; IQR: interquartile range; PFS: progression-free survival; OS: Overall survival; FIGO: International Federation of Obstetrics and Gynecology

**Table 2:** Clinicopathological characteristics of study cohorts and thrombocytosis (*n*=72)

Characteristics	Platelet counts		*P*
	Normal counts (%)	Thrombocytosis (%)	
Age in year, mean±SD	54.3±10.1	55.0±11.6	0.782^a^
Parity, median (IQR)	3.0 (1.0, 4.3)	2.0 (1.0, 2.3)	0.009^b^
BMI in kg/m^2^, median (IQR)	23.8 (19.9, 28.9)	27.3 (22.8, 29.7)	0.052^b^
CA-125 in U/mL, median (IQR)	268.0 (67.0, 541.5)	451.0 (362.4, 1150.8)	0.018^b^
Median PFS (IQR) months	24.0 (13.0, 35.3)	7.5 (6.0, 13.0)	<0.001*^b^
Median OS (IQR) months	36.0 (36.0, 36.0)	21.0 (17.0, 24.0)	<0.001*^b^
Menopausal status			0.341
Premenopausal	17 (51.5)	16 (48.5)	
Postmenopausal	25 (64.1)	14 (35.9)	
Comorbidity			0.963
No	32 (58.2)	23 (41.8)	
Yes	10 (58.8)	7 (41.2)	
Type of primary treatment			0.002
Primary debulking	26 (78.8)	7 (21.2)	
Neoadjuvant chemotherapy	16 (41.0)	23 (58.0)	
Extent of cytoreductive surgery			<0.001*
Optimal	29 (78.4)	8 (21.6)	
Suboptimal	13 (37.1)	22 (62.9)	
Ascites			0.002
No	28 (75.7)	9 (24.3)	
Yes	14 (40.0)	21 (60.0)	
FIGO stage			0.008
Early	16 (84.2)	3 (15.8)	
Advanced	26 (49.1)	27 (50.9)	
Disease grade			0.075
Low grade	15 (75.0)	5 (25.0)	
High grade	27 (61.9)	25 (48.1)	
Histological type			0.011
Serous	23 (47.9)	25 (52.1)	
Non-serous (*n*=10)	19 (79.2)	5 (20.8)	

SD: standard deviation; IQR: interquartile range; PFS: progression-free survival; OS: overall survival; BMI: body mass index.

a Independent sample T-test;

b Mann-Whitney U test;

* *P*: Significant values at 95% confidence interval

**Table 3:** Cox Regression analysis of the clinicopathological factors associated with PFS

Characteristics	PFS		*P*
	Hazard ratio (HR)	95% CI	
Age	0.67	0.23–1.96	0.467
Menopausal status	3.05	0.89–10.52	0.077
Parity	1.13	0.56–2.28	0.738
BMI	0.69	0.30–1.58	0.374
Serum CA-125 levels	0.14	0.03–0.60	0.008
Comorbidity	3.50	0.83–14.87	0.089
Cytoreduction	1.35	0.39–4.71	0.634
Primary treatment	1.28	0.43–3.75	0.658
FIGO stage	152.19	16.55–1399.43	<0.001*
Tumor grade	3.94	0.45–34.50	0.216
Histological type	0.46	0.12–1.79	0.262
Ascites	0.99	0.40–2.41	0.974
Thrombocytosis	0.25	0.83–0.75	0.014

CI: confidence interval; BMI: body mass index; FIGO: International Federation of Obstetrics and Gynecology.

* *P*: Significant values at 95% confidence interval

**Table 4:** Cox Regression analyses of clinicopathological factors associated with 3-year OS

Characteristics	OS		*P*
	Hazard Ratio (HR)	95% CI	
Age	0.62	0.10–3.84	0.737
Menopausal status	2.34	0.32–17.10	0.404
Parity	0.83	0.29–2.41	0.737
BMI	1.45	0.59–3.56	0.419
Serum CA125 levels	0.50	0.04–5.84	0.579
Comorbidity	0.38	0.06–2.47	0.310
Cytoreduction	0.10	0.01–3.07	0.189
Primary treatment	0.89	0.16–4.81	0.891
FIGO stage	1.74	0.43–74.64	0.761
Tumor grade	3.17	0.043–235.40	0.599
Histological type	0.24	0.01–6.01	0.387
Ascites	1.27	0.31–5.26	0.737
Thrombocytosis	0.03	0.01–0.27	0.002

Abbreviations: CI, confidence interval; BMI, body mass index; FIGO, International Federation of Obstetrics and Gynecology.

* *P*: values at 95% confidence interval
